# Gene expression atlas of fruit ripening and transcriptome assembly from RNA-seq data in octoploid strawberry (*Fragaria* × *ananassa*)

**DOI:** 10.1038/s41598-017-14239-6

**Published:** 2017-10-23

**Authors:** José F. Sánchez-Sevilla, José G. Vallarino, Sonia Osorio, Aureliano Bombarely, David Posé, Catharina Merchante, Miguel A. Botella, Iraida Amaya, Victoriano Valpuesta

**Affiliations:** 1 Instituto Andaluz de Investigación y Formación Agraria y Pesquera (IFAPA), Centro de Churriana, Málaga, Spain; 2 Departamento de Biología Molecular y Bioquímica, Instituto de Hortofruticultura Subtropical y Mediterranea (IHSM), Universidad de Málaga-Consejo Superior de Investigaciones Científicas, Málaga, Spain; 3 Department of Horticulture, Virginia Tech, 24060 Blacksburg, VA USA

## Abstract

RNA-seq has been used to perform global expression analysis of the achene and the receptacle at four stages of fruit ripening, and of the roots and leaves of strawberry (*Fragaria* × *ananassa*). About 967 million reads and 191 Gb of sequence were produced, using Illumina sequencing. Mapping the reads in the related genome of the wild diploid *Fragaria vesca* revealed differences between the achene and receptacle development program, and reinforced the role played by ethylene in the ripening receptacle. For the strawberry transcriptome assembly, a *de novo* strategy was followed, generating separate assemblies for each of the ten tissues and stages sampled. The Trinity program was used for these assemblies, resulting in over 1.4 M isoforms. Filtering by a threshold of 0.3 FPKM, and doing Blastx (E-value < 1 e-30) against the UniProt database of plants reduced the number to 472,476 isoforms. Their assembly with the MIRA program (90% homology) resulted in 26,087 contigs. From these, 91.34 percent showed high homology to *Fragaria vesca* genes and 87.30 percent *Fragaria iinumae* (BlastN E-value < 1 e-100). Mapping back the reads on the MIRA contigs identified polymorphisms at nucleotide level, using FREEBAYES, as well as estimate their relative abundance in each sample.

## Introduction

Cultivated strawberry (*Fragaria* × *ananassa*) is a hybrid octoploid species (2n = 8x = 56) that resulted from a spontaneous cross of two wild octoploid species, *F. chiloensis* and *F. virginiana* ^1^. Recently, dissection of its genome, after deep sequencing of wild relatives, led to the generation of a virtual reference genome for this species and helped to establish a subgenomic structure^2^. However, the whole-genome sequence of *F*. × *ananassa* has not been published. In the absence of a sequenced genome, it is appealing for researchers to use information from a full-length transcriptome that, in addition to the sequence information, could provide gene expression data. This possibility is facilitated by RNA sequencing (RNA-seq) technology, which provides a comprehensive profile of a transcriptome, in the experimental conditions under study.

The wild strawberry *Fragaria vesca* is a diploid that is the donor of one of the four subgenomes of the octoploid species^3–5^. *F. vesca* has been considered a model species not only for the *Fragaria* genus but also within the Rosaceae family^6^. The genome of *F. vesca* has been sequenced^7^, and more recently, a new assembly of this *Fragaria* reference genome has been released, which includes 208.9 Mb of scaffold sequence assembled in seven pseudochromosomes and 1.9 Mb remaining unanchored^3^. In addition, a continuous annotation updating the *F. vesca* genome by the NCBI is in progress and other re-annotation efforts have been published^8^. The availability of this sequence information has been critical to the performance of global expression analysis by RNA-seq in this species^9,10^, which has shed light on key developmental processes occurring in the fruits of this species at early stages of development. Fruit transcriptome and regulation of transcript expression during ripening of cultivated strawberry probably differ from that of *F. vesca* due to aspects related to its polyploid nature and also due to differences between these species developmental characteristics such as fruit size, firmness and aroma. Therefore, there is a need for basic information on genes specifically expressed during fruit development and ripening of cultivated strawberry. The availability of the *F. vesca* genome and its high macrosynteny with the octoploid genomes offer the possibility of mapping the transcriptomic data of *F*. × *ananassa* to a reference genome, and eventually facilitate the identification of key regulators of fruit ripening, such as transcription factors and plant hormones. The *F. vesca* genome has been used for the deep analysis of the fruit transcriptome of less closely related species, such as *Rubus* sp^11^.

In previous studies on strawberry, global analyses of the fruit development process have been performed at metabolomic^12^, proteomic^13,14^ and transcriptomic levels^15,16^. Transcriptome analysis not only provides information about sequences and the abundance of transcripts, but can also detect polymorphisms among transcripts corresponding to the same gene. This is especially relevant for *F*. × *ananassa*, an octoploid species with high levels of heterozygosity^17^.

Three strategies can be used to assemble a transcriptome when a reference genome is available: reference-based, *de novo*, or a combination of the two^18^. The reference-based method is the most straightforward, but it requires a high-quality reference genome. In strawberry, the *F. vesca* genome can be used as a reference for cultivated *F*. × *ananassa*; however, it still presents a significant amount of unanchored scaffolds and gene annotation is incomplete. *De novo* assembly of a complex transcriptome from short reads presents additional challenges, such as the identification of a number of sequence variants, which are expected in a heterozygous and allopolyploid species such as *F*. × *ananassa*. However, it has some benefits^18^, as it is independent from the quality of the reference genome, and it allows for the detection of transcripts specific to the cultivated strawberry or those with low similarity between the two species. However, a previous study the allohexaploid *Avena sativa*, identified a significant number of homoeoalleles and paralogs in the species starting from a RNA-seq dataset^19^. Different *de novo* assemblers from RNA-seq data have been tested in species with sequenced genomes, such as *Arabidopsis* and rice^20^. In this comparison, the Trinity program offers a good balance of expected achievements, including the identification of variants in the transcript isoforms. This program was originally developed for the assembly of transcriptomes of fission yeast, mouse and whitefly, whose reference genomes were not yet available^21^.

In order to analyse specific changes in transcript expression between the achenes and the receptacle during strawberry ripening, we have performed a global expression analysis by RNA-seq of the two organs separately, at four stages of strawberry fruit ripening. To allow further comparisons with vegetative tissues, leaf and root transcriptomes have also been included in our analysis. We have used the most updated version of the *F. vesca* genome^3^ (Fvb_genome) to map the reads and obtain expression profiles. Global analysis of the transcriptomic data identified key processes associated to specific tissue/developmental stages. Thus, the involvement of ethylene in the receptacle ripening has been reinforced^22^, after analysis of the *ETHYLENE RESPONSE FACTOR* (*ERF*) gene family. In addition, *de novo* transcriptome assembly was performed at a tissue/stage level by employing the Trinity program and the MIRA program to generate contigs, whose final number was close to the number of genes predicted for *F*. × *ananassa* genome^2^. The polymorphism was analysed after mapping back the reads on the contigs, with the FREEBAYES program. The analysis gives information on the nucleotide variations in the assembled contigs sequences, at the tissue/stage level.

## Results

### **Mapping of RNA seq reads in the*Fragaria vesca*genome**

**Figure 1:**
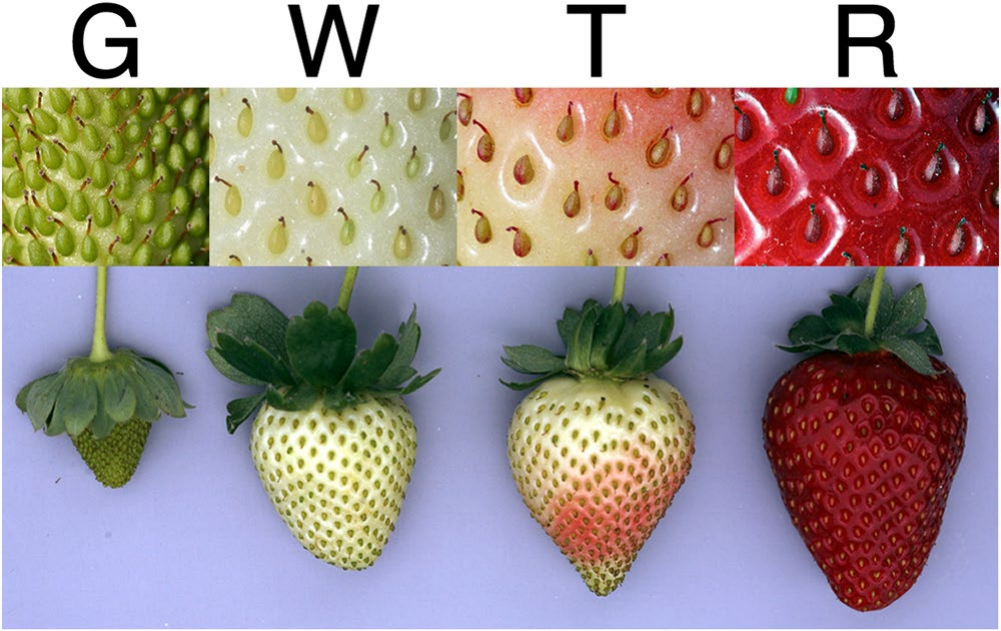
Images of representative strawberry fruits corresponding to the four developmental stages selected for RNA-seq expression. Green (G), White (W), Turning (T), and Red (R).

To obtain a comprehensive picture of the strawberry fruit transcriptome we selected four representative stages of development to perform the expression analyses by RNA-seq. This analysis was performed separately in the achenes and receptacles. This separation is critical for dissecting the different gene networks operating in these two organs during the ripening process. The four sampling times were defined by the colour of the receptacle as green (G), white (W), turning (T), and red (R) (Fig. 1). To explore differential and specific gene expression between fruits and vegetative organs, leaf and root samples were included in the study.

**Figure 2:**
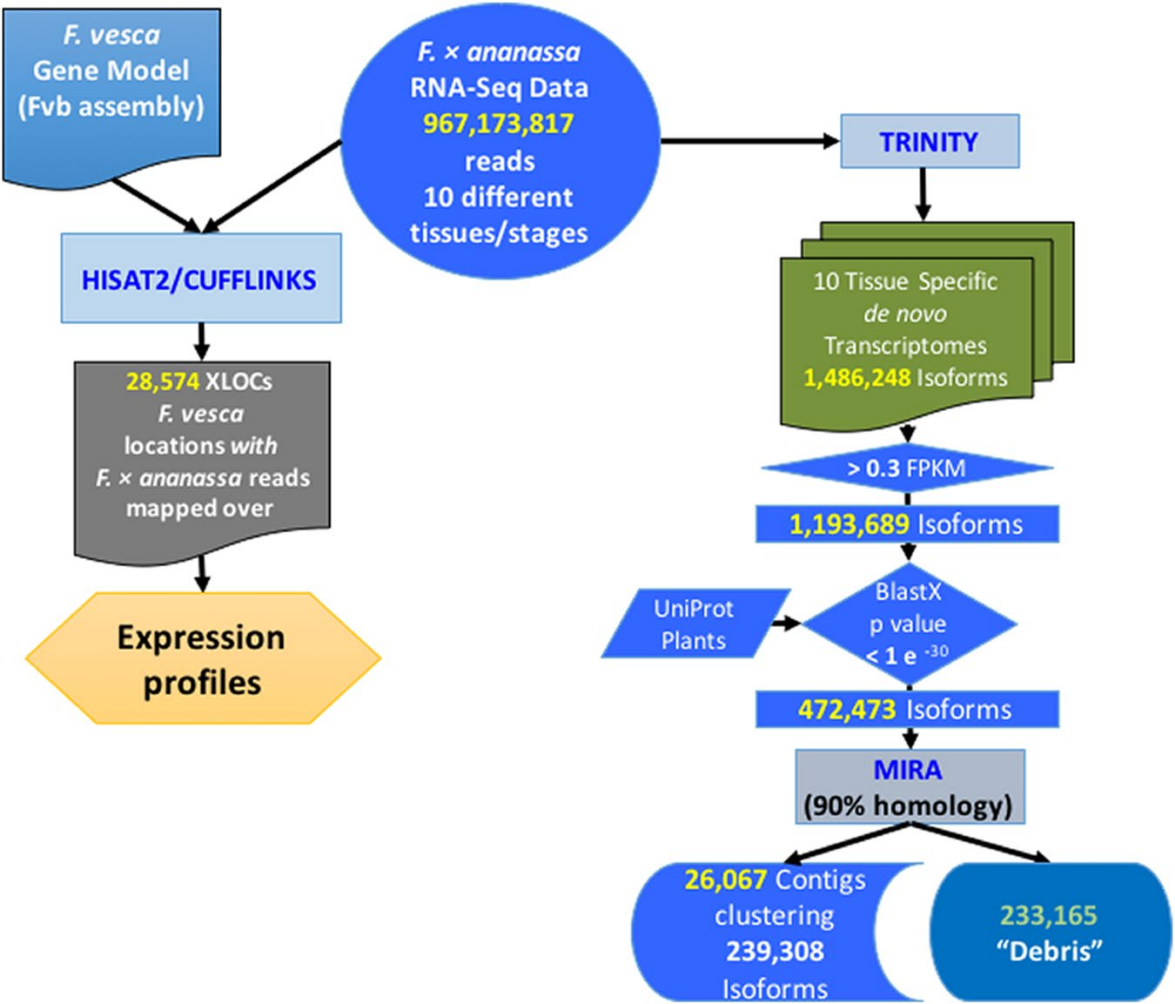
Outline of the steps followed in the analysis of the RNA-seq data to obtain information on the gene expression profiles, and the *de novo* generation of the transcriptome by sample, and its assembly in contigs.

Given the high overall nucleotide sequence identity between the homologous genes of *F. vesca* and *F*. × *ananassa* ^23^, the reads were initially aligned against the *F. vesca* genome (Fvb_genome)^3^ (Fig. 2). Over 967 million paired reads (PRJEB12420, http://www.ebi.ac.uk/ena) were aligned in total, ranging from 84 million in turning achene to 128 million in red receptacle (Supplementary Table S1). After processing the raw sequences, as described in the Methods section, an average of 77.5% were mapped per sample to the *F. vesca* genome (Fvb assembly^3^), with minor differences among samples (Supplementary Table S1). This value can be considered satisfactory because it is known that TopHat/Hisat2 can achieve a similar percentage of short reads mapped in an assembled and annotated homologous genome^24^. The merged number of locations mapped from the ten samples was 28,574 (XLOCs) (Fig. 2, Supplementary Table S1). From these, 25,490 (89%) corresponded to NCBI *F. vesca* RefSeq annotations, and 3,084 (11%) to new mapping coordinates. The values obtained for mapped reads, 77.5% on average, and the number of putative genes targeted in the *F. vesca* genome, 89%, support the validity of the RNA-seq data from *F*. × *ananassa* and the reliability of its analysis after mapping onto the model *F. vesca* genome. Among samples, green achene was the sample with the highest number of expressed genes, followed by roots and leaves (Supplementary Table S1). In both the achene and the receptacle, the total number of expressed transcripts decreased during ripening, with red achenes (17,918) and red receptacles (16,509) having the lowest number of expressed genes compared with the rest of tissues (Supplementary Table S1).

**Figure 3:**
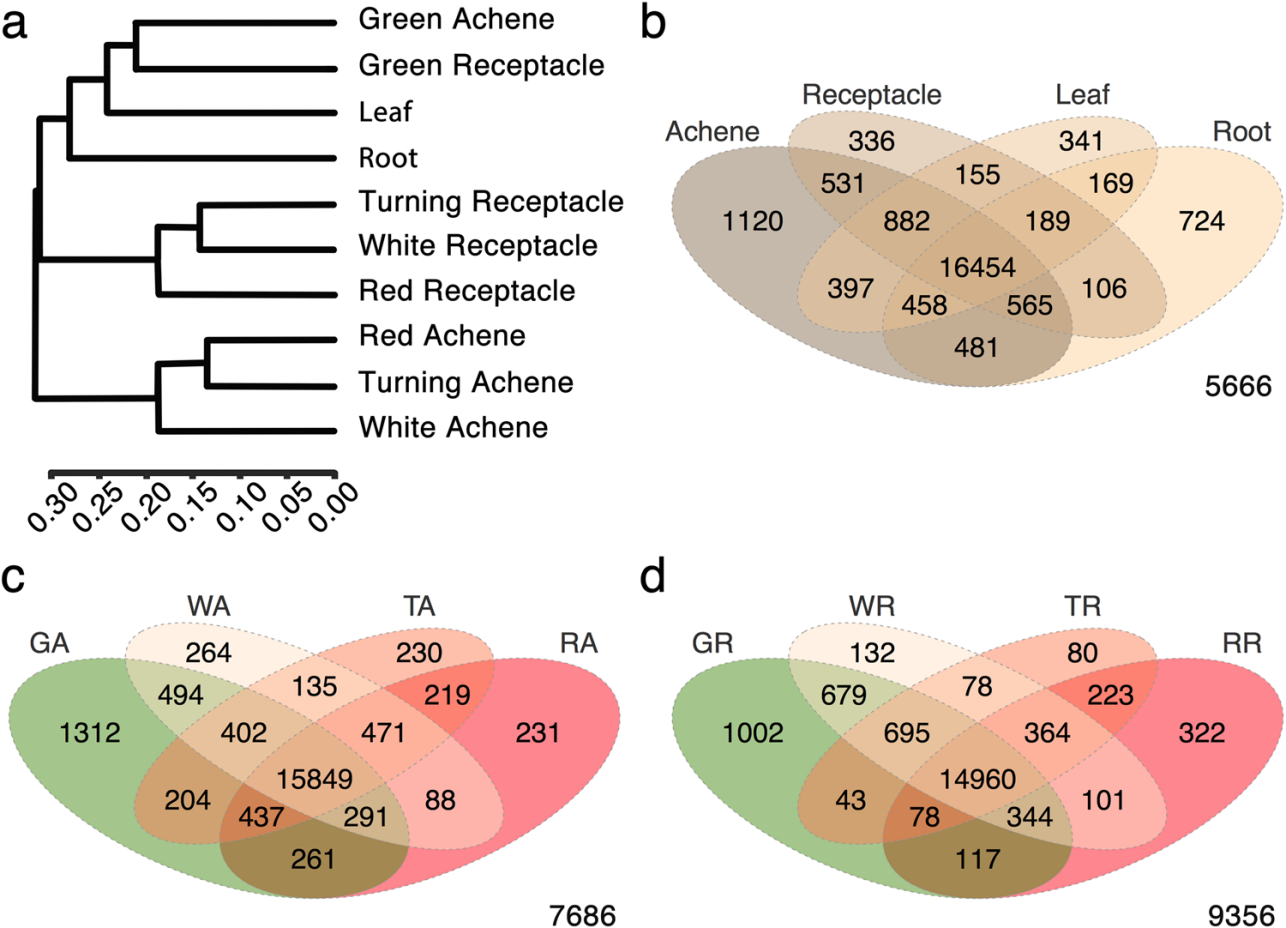
Global analysis of the RNA-seq expression data, with values higher than 0.3 FPKM, in the ten samples: achene and receptacle at four developmental stages each, leaf, and root. (**a**) Dendrogram representing the clustering of the samples based on the gene expression levels (CummRbund v2.7.2, an R package). (**b**) Venn diagram representing the number of genes expressed per sample, and their intersection with other samples. Analysis was performed for the four tissues. (**c**) and (**d**) Venn diagrams representing the number of genes expressed for achene and receptacle separately, at the four developmental stages.

Clustering analysis of the genes expressed, with values higher than 0.3 FPKM, in the ten different samples showed that the achene and receptacle at the green stage were more similar to leaf tissue in their transcriptome, and were also grouped more distantly with roots (Fig. 3a). This result most likely reflects the prevalence of photosynthetic and growth-associated activities of the achene and receptacle at the green stage over other developmental events, making them transcriptionally closer to vegetative tissues. During the green stage, there is a close connection between the achene and receptacle, mainly mediated by auxin^23^ and this is probably mirrored in their global transcriptional activity. After the green stage, the achene and receptacle samples group separately (Fig. 3a). Interestingly, the transition from the white to red stage appears different in these two parts of the fruit. In achenes, the major changes in genes expression occur from the white to turning/red transition, whereas in receptacle, the differences in the transcribed genes are higher in the transition from white/turning to red. Whether these global transcriptional analyses are indicative of independent timely elapsed developmental programs for these two organs should be investigated, as they may facilitate the understanding of the growth and ripening of the strawberry fruit.

Comparisons of the transcriptomes of each sample revealed that the highest number of organ-specific genes corresponds to the achene transcriptome (1,120), more than three times the number of receptacle (336), followed by the root, with a total of 724, while the leaf shows 341 tissue-specific genes (Fig. 3b). The achenes are the true fruits of the strawberries, and the higher number of achene-specific genes over those of the receptacle may reproduce the more complex structure of that organ^25^. In the achenes, the highest number of stage-specific transcripts corresponded to the green stage (1,312) (Fig. 3c). At this time (Fig. 1), the achenes are at the final stages of embryo and cotyledons development^25^. Detailed dissection of the achene development based on transcription analysis has been previously performed in *F. vesca* ^9^. In this study, it was reported that ghosts and embryos also presented the highest number of tissue-specific genes in wild strawberry at early stages of fruit development. At the red stage, processes occurring in the achene are associated with seed coat development and preparation for dormancy, with a significantly lower number of stage-specific transcripts (231). Similarly, the highest number of stage-specific genes was also found at the green stage in the receptacle (1,002), followed by the red stage (322) (Fig. 3d). The turning receptacle had the lowest number of stage-specific genes (80), which might suggest that this stage represents a transition from white to red developmental stages, rather than a specific developmental stage. However, detailed analysis of these genes would be needed to support this conclusion. In the receptacle, the number of genes that were shared between green and white stages was 679, while only 78 were shared between white and turning, and 223 between turning and red. Whether these numbers are indicative of milestones in the strawberry receptacle development need to be analysed in detail, jointly with the values obtained for differentially expressed genes (DEGs).

**Figure 4:**
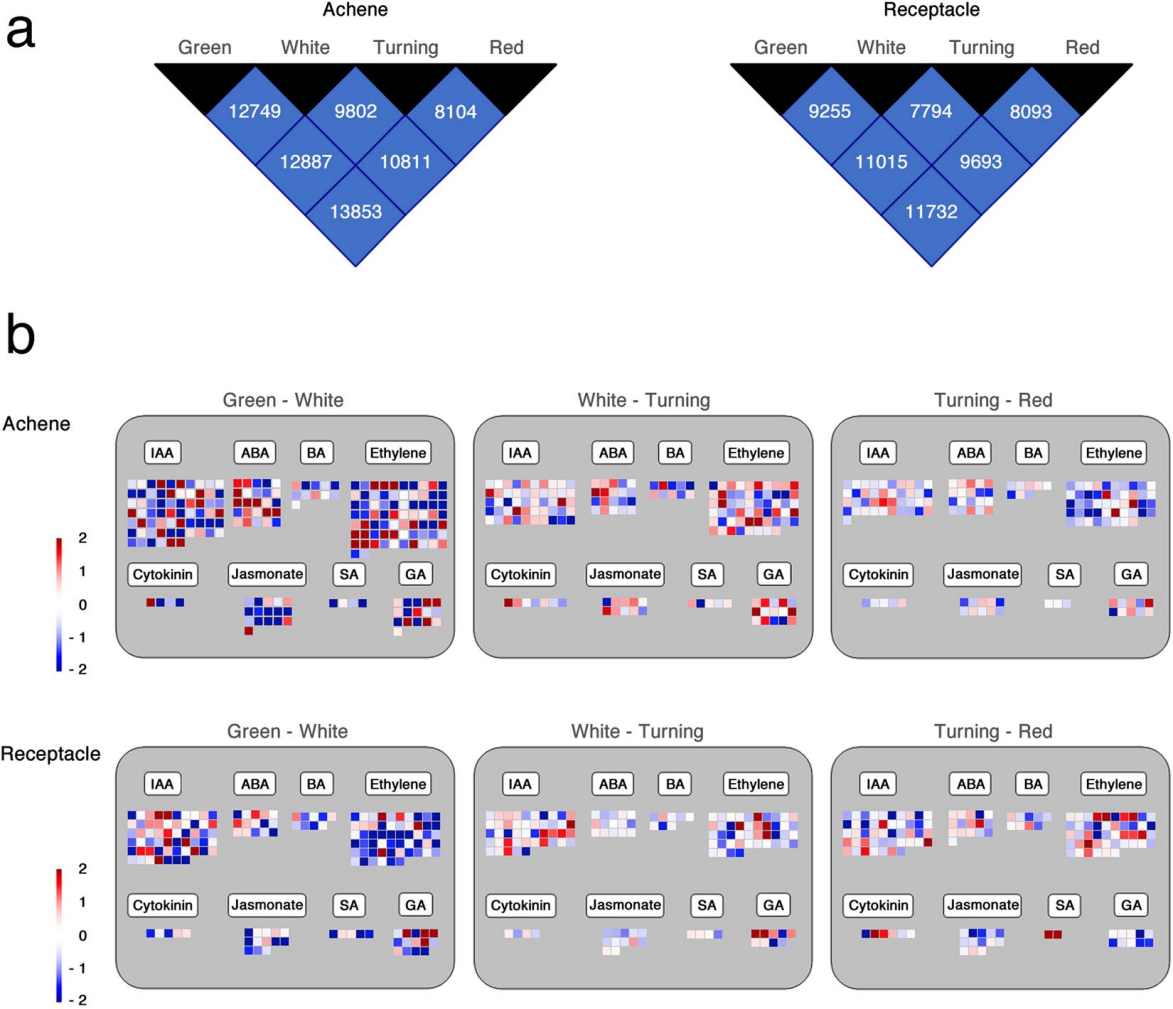
(**a**) Summary of the number of genes differentially expressed (DEG) between two stages of development of achene and receptacle. (**b**) Regulation overview corresponding to the DEGs in the successive transitions from green (G), white (W), turning (T) and red (R), in the achene (A) and the receptacle (R), obtained by MapMan analysis. The color key indicates the log2 (fold change) between the transitions.

Analysis of DEGs between the different stages of achene and receptacle development (Supplementary File S1) is showed in Fig. 4a. In general, number of DEG between two stages were higher for achene, probably reflecting its more complex composition. In both organs, the highest number of DEGs between two consecutive stages were found in the transition from green to white stage, probably associated to the decline of the photosynthetic activity. Overview of the metabolism changes in the achene and receptacle from green to red stages, made by MapMan analysis, shows that a continuous decrease in the expression of genes corresponding to light reactions and Calvin cycle (Fig. S1). However, DEGs associated to primary metabolism (carbohydrates, tricarboxylic acid cycle, and mitochondrial electron transport) displayed a different pattern for achene and receptacle, clearly decreased in achene, with highest changes in the transition from white to red, while changes in receptacle slightly increased from green to red stages. Also, in secondary metabolism there is some asynchrony in the DEGs at different transitions between the achene and the receptacle.

Regulation overview by MapMan of DEGs in ripening achene and receptacle includes genes related to different plant hormones. Among these, auxin and ethylene (Files S2-S7) represent the two major groups (Fig. 4b). Recently, the analysis of auxin in the ripening process of the receptacle has been reported^26^. Regarding ethylene, the *ERF* family, as a representative family of regulatory genes for his hormone, is here studied in more detail.

### The ERF family

**Figure 5:**
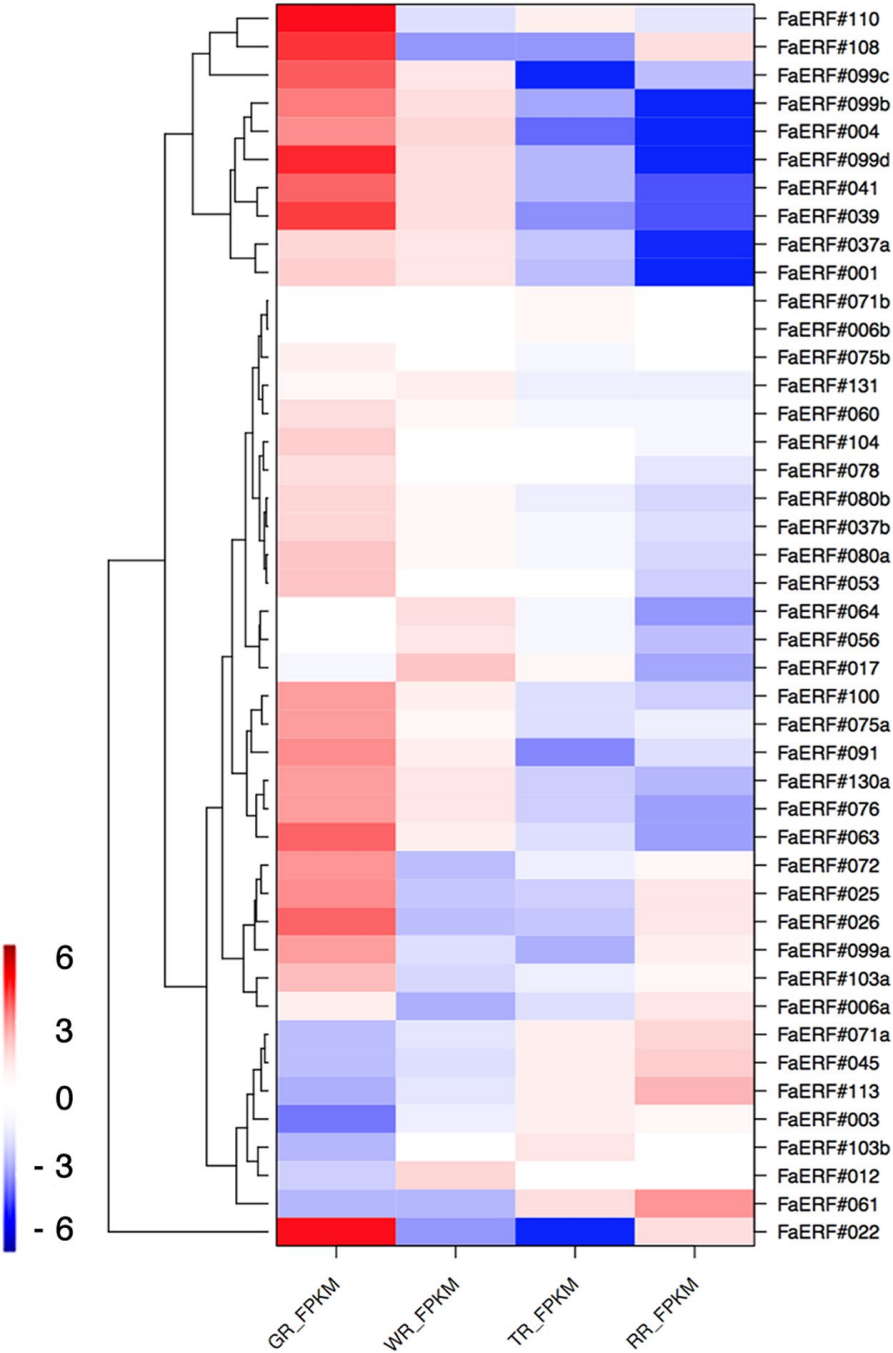
Heatmap representing the *FaERFs* expression profiles in the receptacle at the four sampled stages: green, white, turning, and red. For each gene, the relative FPKM value was calculated (FPKM^tissue^/FPKM^median^), and the Log_2_ (fold change) scaled expression FPKM values were used to perform hierarchical clustering. The levels of relative decreased expression (blue) or increased expression (red) are shown for each receptacle developmental stage.

**Figure 6:**
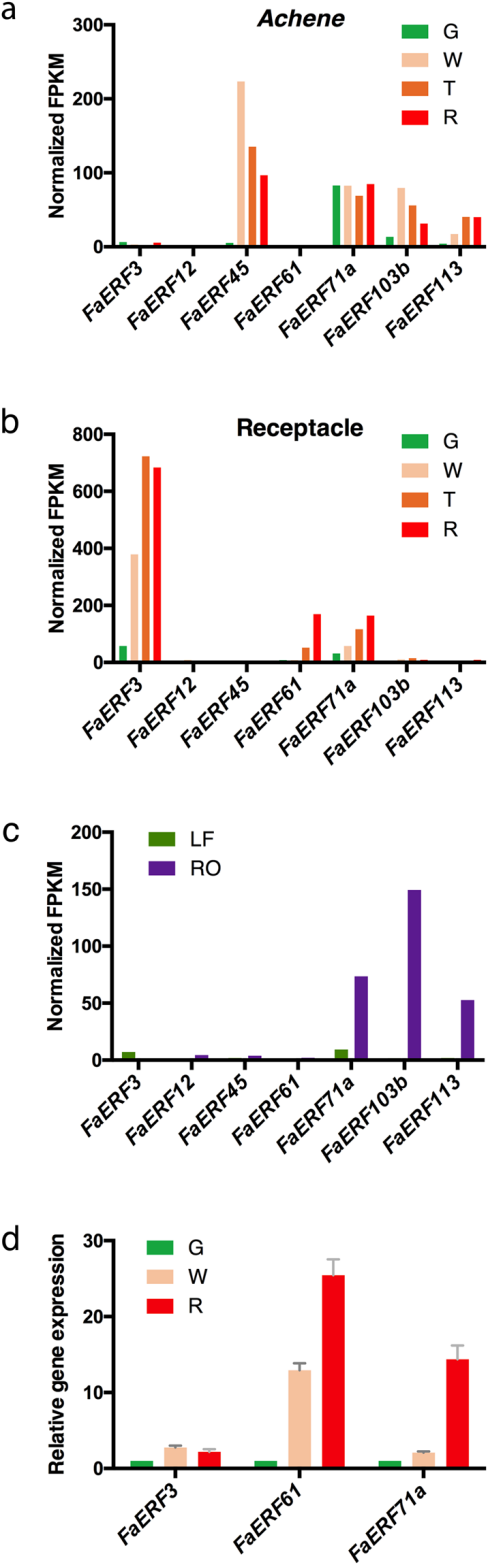
Expression profile by RNA-seq (**a–c**) and q-PCR (**d**) of selected *FaERF* genes. Expression by RNA-seq is expressed in normalized FPKM for achene (**a**) and receptacle (**b**) at four developmental stages, and leaf and root (**c**). Relative expression by q-PCR of *FaERF3*, *FaERF61* and *FaERF71a* correspond to receptacle at three developmental stages. Error bars indicate ± SE of three biological replicates. Different samples are represented as Green (G), White (W), Turning (T), Red (R), Leaf (LF), Root (RO).

Ethylene response factors (ERF), jointly with AP2 and RAV families of TFs, constitute the superfamily of AP2/ERF TFs^27^. Our analysis here is restricted to genes of the *ERF* family, as some of them are part of the ethylene signalling pathway^28^. The analysis of the *FaERF* family was restricted to the receptacle because we had previously found clear changes in the expression of other genes in this organ associated with ethylene action^22^. In *F. vesca*, a total of 96 members of the *ERF* family were identified^9^. Reads of the RNA-seq expression data in the receptacle mapped on 45 of these genes, with expression values higher than 1.0 FPKM. Alignment of the sequences of the mapped *F. vesca* genes with the *Arabidopsis* genes (www.arabidopsis.org, May 22, 2016) (Supplementary Fig. S2) was used to name the strawberry genes. Clustering analysis of the expression patterns of the strawberry *ERFs* in the developing receptacle are shown in Fig. 5. Most of the *ERF* genes display higher expression levels at the green than at the red stage. However, a cluster of seven genes in the lower part (*FaERF71a*, *FaERF45*, *FaERF113*, *FaERF3*, *FaERF103b*, *FaERF12*, and *FaERF61*) showed up-regulation during ripening of the receptacle. In tomato, 19 of the total 77 *ERFs* in the genome show differential expression with fruit ripening^29^. The expression values of the seven *FaERF* genes in the ripening receptacle, as well as in other samples, are shown in Fig. 6. Three of these genes (*FaERF3*, *FaERF61* and *FaERF71a*) show a high expression level of over 100 FPKM in the ripe receptacle (Fig. 6b). For two of them, *FaERF3* and *FaERF61*, their expression in other organs and tissues was very low (Fig. 6a,c). The *FaERF71a* gene, albeit presenting the highest expression in receptacle (Fig. 6b), was also expressed in achene and root at significant level (Fig. 6a,c). The increased expression with ripening in receptacle was confirmed by q-PCR for these three genes (Fig. 6d).

From the information provided here (http://www.ebi.ac.uk/ena, Ref. PRJEB12420), the analysis can be extended to other gene families. However, because *F*. × *ananassa* is a highly heterozygotic octoploid species^20^, information on allelic expression for each gene would be of great interest. The *de novo* assembly of the *F*. × *ananassa* transcriptome from the RNA-seq data would provide information on allelic gene expression.

### *De novo* transcriptome assembly

Our design of the RNA-seq experiment included a representative variety of tissues and stages (Fig. 1) at a high sequencing depth (a minimum of 84 million reads/sample, Supplementary Table S1). For the assembly, we chose the *de novo* strategy rather than the reference-based strategy^18^. The *F. vesca* genome sequence, while being continuously updated^7^, still represents a draft in which a significant amount of sequence is still unanchored, and/or incorrectly assembled when compared to genetic maps^3,7^. In addition, an assembly based on a reference genome from a different species, although closely related, would inevitably miss transcripts from divergent genes^18^. A main goal in the present study was the identification of sample-specific transcripts. In a polyploid species such as *F*. × *ananassa*, this objective would eventually facilitate the identification of the prevailing alleles expressed in each sample. Therefore, the reads obtained from the ten different samples were analysed separately with the Trinity program^21^, using the default parameters.

The total number of isoforms obtained was 1,486,248 (Fig. 2), with an N50 value of 1,526 and an average length of 821 bp. Isoforms with values for normalized reads lower than 0.3 FPKM were not considered for analysis, as they corresponded to genes too lowly expressed^9^. Then, the number of assembled transcripts using Trinity was reduced to 1,193,689. These sequences were filtered by a BlastX sequence homology search using the UniProt Plants Database, setting the highest limit for the E-value at 1e-30. The objective was to remove data resulting from contaminations of the samples with mRNA from other organisms and to specifically focus on plant genes. The number of sequences was reduced to 472,473 (Supplementary Files S8). These isoforms represent putative transcripts assembled separately in the ten different samples. Therefore, it was expected that a number of them could be the result of the expression of the same gene in different samples. Moreover, because *F*. × *ananassa* is an octoploid with a high level of heterozygosity, some of the transcripts could be the result of the expression of different alleles (homologs and homoeologs) of the same gene. Thus, an additional analysis of the Trinity isoforms and their assembly into contigs was performed to advance the characterization of the *F*. × *ananassa* transcriptome. The program selected for this task was MIRA because this program is an assembler that has been successfully used to reconstruct mRNA transcript sequences gathered in EST sequencing projects^30^.

**Figure 7:**
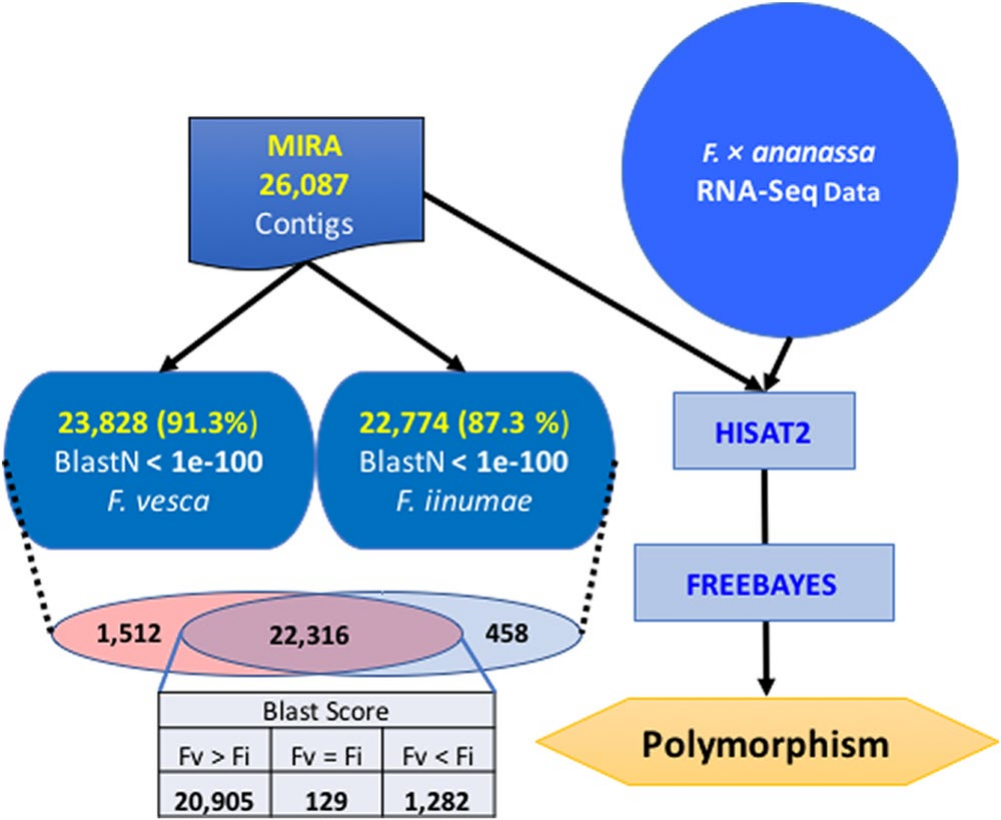
Outline of the steps followed in the analysis of the contigs generated by the MIRA program, and the search of their polymorphism after mapping back the RNA-seq data on them.

*De novo* assembly in highly polymorphic regions using assemblers based on De Bruijn Graphs, as in Trinity, might yield a higher number of contigs than the actual number of genes expressed, thus producing a redundant and fragmented transcriptome^31^. Out of the initial 472,476 isoforms, the program considered 233,165 sequences as “debris.” The vast majority of these “debris” correspond to chimeras generated by Trinity in the assembly process (213,299). In general, assembly of sequences from deep sequencing data in polyploidy species produces a high number of chimeras, with Trinity program having a slightly superior success^32^. The rest of debris corresponds to transcripts not assembled, due to a lack of overlap (5,731) or clustering in tiny-cluster-orphans (10,795). The remaining 239,308 sequences (50.65% of the Trinity isoforms) were assembled in 26,087 contigs (Fig. 7, Suppl. File S9). These contigs, assembled by the MIRA program from the isoforms produced by the Trinity program, represent the *F*. × *ananassa* transcriptome. Since the total number of contigs, 26,087, is close to the total number of *F. vesca loci* mapped with reads in the previous approach (28,574 XLOCs, Fig. 2), most probably the four subgenomes of *F*. × *ananassa* have been collapsed.

To characterize this set of contigs, further analysis of homology to known nucleotide sequences of *Fragaria vesca* (NCBI database) and *Fragaria iinumae* ^2^ was performed by BlastN with the limit of an E-value of 1e-100, which identifies homologous genes in these species at high stringency. It was found that 24,286 of the contigs were represented in the genome of these two species, with 23,828 in *F. vesca* and 22,774 in *F. iinumae* (Fig. 7). From these contigs, 22,316 were common to both species, and 1,512 were represented only in the *F. vesca* genome while 458 were only detected in the *F. iinumae* genome. In addition, a total of 1,801 contigs were not represented in the genomes of these two species. Analysis of the contigs represented in the two species showed that most of them, 20,905 contigs, displayed a higher B-score to the *F. vesca* gene than to *F. iinumae*, while the B-score for 1,282 genes was higher for *F. iinumae* (Fig. 7). This analysis cannot be used to infer conclusions about the genomic structure of *F*. × *ananassa* because it most likely reflects the quantity and the quality of the genomic information available for the two diploid species.

### Identification of tissue/stage-associated polymorphisms in the transcriptome

Single contigs in a *de novo* assembled transcriptome include a variable number of transcripts whose sequence variability, even small, could be collapsed. Highly similar transcripts, from different alleles or homoeologs, are likely to be assembled into a single contig and will require additional post-assembly steps to resolve^18^. Mapping back the RNA-seq reads on the MIRA contigs (Fig. 7) led to an overall alignment of 69.86%, ranging from 59.11% in the turning achene sample to 76.88% in the red receptacle (Supplementary Table S2). This value is slightly lower than the percentages obtained after mapping the reads onto the *F. vesca* genome (Supplementary Table S1). As a result of this mapping, the FREEBAYES program allows the identification and estimation of polymorphism frequency in the reads of *F*. × *ananassa* here studied, as well as their association with the different samples/stages. As a proof of concept, we present the results obtained from applying this analysis to the *FaERF71a* gene (Fig. 6).

**Figure 8:**
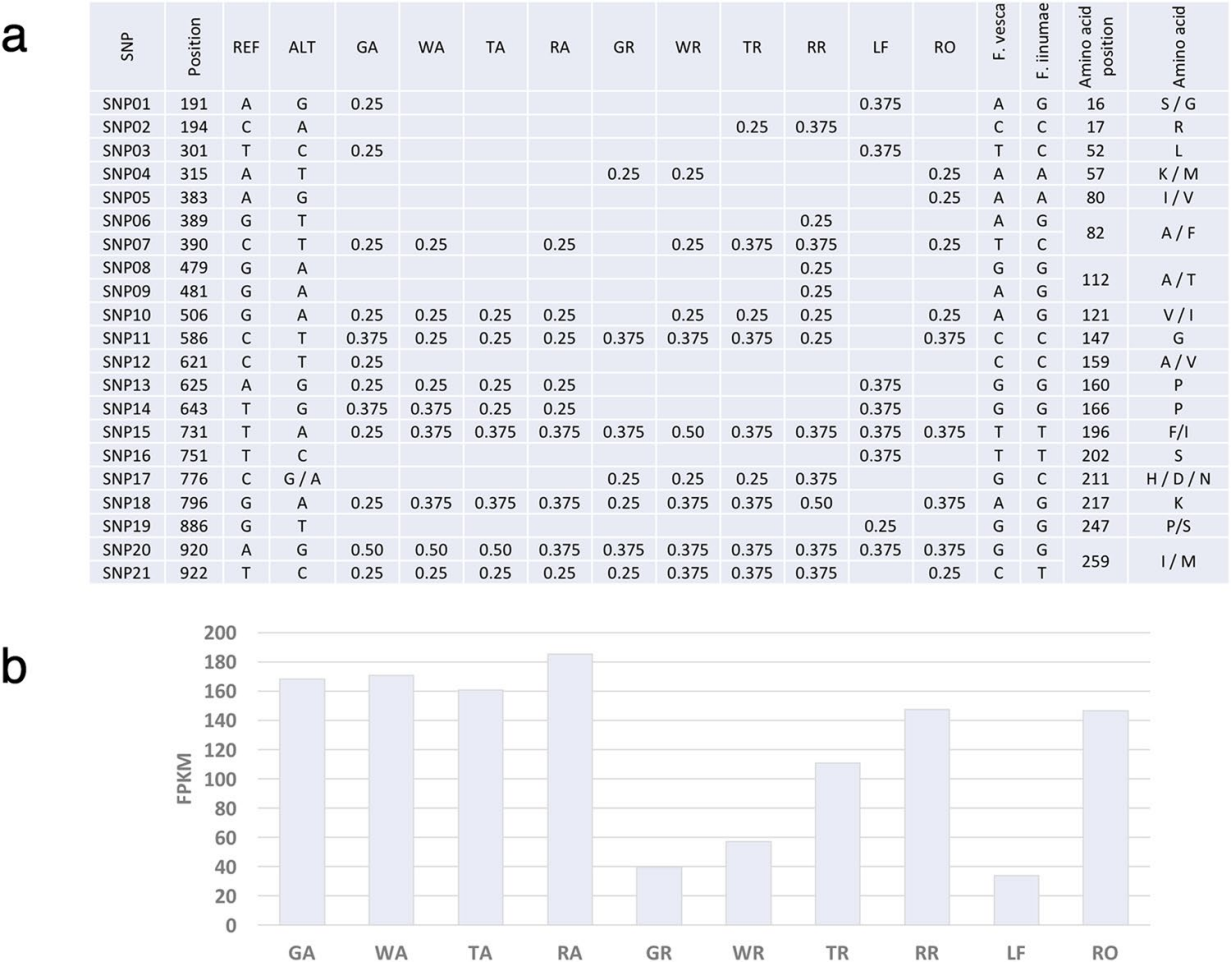
(**a**) Summary of the polymorphisms identified in *FaERF71a* by FREEBAYES after mapping back the RNA-seq reads on the MIRA contigs. At each position the most abundant nucleotide (REF) and the alternative(s) (ALT) are indicated. Frequency for the alternative nucleotide at each position, in every sample, as estimated by FREEBAYES is shown. The corresponding nucleotide of *F. vesca* and *F. iinumae* is indicated at each position, as well as the amino acid involved and the alternative due to the nucleotide change. Samples correspond to green achenes (GA), white achenes (WA), turning achenes (TA), red achenes (RA), green receptacle (GR), white receptacle (WR), turning receptacle (TR), red receptacle (RR), leaf (LF) and root (RO). (**b**) Expression profile of the *FaERF71a* gene in the different tissue/stage samples resulting from mapping back the RNA-seq reads on the MIRA contigs.

The *FaERF71a* gene presents the highest similarity to the *F. vesca* gene 07057 (located in the *F. vesca* genome at LOC101293287, gi|764572142), and corresponded to the MIRA contig 1900 (Suppl. File S2). The polymorphisms found by FREEBAYES after mapping the RNA-seq reads on this contig are shown in Fig. 8a. A total of 21 positions were identified as polymorphic, with one position presenting three alternative nucleotides. The sequences of the corresponding genes in two diploid ancestors of *F*. × *ananassa* ^2,3^, such as *F. vesca* and *F. iinumae*, are known, and the nucleotides in the polymorphic sites are also shown in Fig. 8a. In 9 of the 21 polymorphic sites, the nucleotides were different between the two diploid *Fragaria* species. Interestingly, in 3 of these positions, the more abundant nucleotide corresponded to *F. vesca* while in the other 9 positions, the most abundant *F*. × *ananassa* nucleotide matched those of *F. iinumae*.

The FREEBAYES analysis also gives the percentage of a specific nucleotide per position for each sample. It was found that there were different SNP frequencies among samples in the majority of the polymorphic positions. The samples with higher number of polymorphic positions were the green achene and the red receptacle, with 12 SNPs each. There were also clear differences in SNP frequency at several positions among the different samples (Fig. 8a). At some positions, the SNP involves a change in the encoded amino acid. This was found in 11 out of the 21 polymorphic positions. Although, in most cases it is a conservative change, there are six positions (amino acids 16, 57, 82, 196, 211, 247) where the amino acid might have structural consequences. Moreover, one of these changes (amino acid position 121) is located in a conserved domain for the gene family, such as the AP2/ERF domain that it is critical for protein function in the ERF family^27^. Mapping back the RNA-seq reads on the MIRA contigs gives the expression value, as FPKM, for each contig. Developmental expression profile for the contig corresponding to *FaERF71a* is shown in Fig. 8b. Although the values are higher than those obtained after mapping on the *F. vesca* genome (Fig. 6a,b), the profile along fruit development was almost identical. This similarity was a general behaviour in all the genes tested. Higher values for FPKM in mapped contigs are possibly related to their smaller size in comparison with the *F. vesca* genes, as result of the assembly process. As indicated above, the challenge is to join the different alternatives of the polymorphic sites (Fig. 8a) in haplotypes, which jointly with the expression level of the gene at each stage (Fig. 8b), will provide valuable information on the different alleles/homoeologs expressed by tissue and developmental stage.

## Discussion

The selection of samples in the study of the strawberry fruit transcriptome appears as a critical factor since it comprises two different organs, achene and receptacle, which differ in their cellular structures and biological functions. This means that their changing transcriptome along ripening can be very different. This is confirmed in the global analysis of DEGs by Mapman. Thus, there is an asynchrony between the achene and the receptacle in the expression of genes of the carbohydrate metabolism and respiration, with an earlier and deeper decrease in the achene with the ripening process. A previous transcriptomic study in the whole strawberry fruit reported a decrease in the oxidative phosphorylation with ripening^16^, that probably reflected the changes taking place mostly in the achene. The occurrence of asynchrony between achene and receptacle in secondary metabolism is somehow expected since the cellular composition of these two organs is very different, as it has been previously reported^15^.

The interplay of hormones in the developmental programs of fruits, including ripening, is well documented. In strawberry, the involvement of some hormones such as auxin^26^, abscisic acid^33^, gibberellin^34^, ethylene^22^, and jasmonic acid^35^ have been reported. Our global analysis of the transcriptomic changes in achene and receptacle along ripening shows a high number of DEGs connected to auxin and ethylene metabolism. While transcriptomic changes in relation to auxin synthesis and signaling of auxin has been analyzed in ripening receptacle^26^, the study is here extended to ethylene. Previously, we reported that ethylene is involved in some of the changes occurring in ripening receptacle^22^. The analysis of the *ERF* family, as key elements in the ethylene signaling pathway^28^, identified a set of three genes with significant expression level in the receptacle (*FaERF3*, *FaERF61*, *FaERF71a*), and an up-regulated expression with ripening. A BLAST search of *FaERF71*a in other species resulted in the highest E-value for the *Arabidopsis RAP2.3* gene (*At3g16770*) and the tomato *SlERF.E1* gene. The RAP2.3 protein interacts with the DELLA protein GAI, which connects the gibberellin (GA) hormone with the activity of ERF transcription factors^36^. This is an interesting finding as a prominent role for GA in the strawberry is receptacle development^34^. Moreover, the GA effect in receptacle development is mediated by the transcription factor *FaGAMYB* ^37^, and the silencing of this gene was accompanied by a decreased expression of *FaERF71a* ^38^. A recent study on the strawberry fruit transcriptome around the ripening process also showed that GA activity preceded ethylene in the fruit ripening process^16^. In tomato, the *SlERF.E1* gene is one of the three *ERF* genes proposed to be important in controlling fruit ripening via ethylene, and also through RIPENING INHIBITOR (RIN)/ NONRIPENING NOR-mediated mechanisms^29^. In contrast, the tomato gene with highest similarity to *FaERF3* is *SlERF.H1*, which was not functionally associated with fruit ripening in this species^29^. However, in grape, the gene with the highest similarity to *FaERF3* is *VvERF46*, which has also been associated with the ripening of the flesh tissue in the fruits of this species^39^. Regarding *FaERF61*, neither the corresponding tomato (*Solyc08g082210*.2) or grape genes have been associated with fruit ripening^27,29^. The corresponding Arabidopsis gene (*At1g64380*) is classified in Group 1 in this species^40^, whose members have no specific function assigned, although it has been reported to be drought-responsive^41^, The transcriptional analysis of the *FaERF* family in ripening strawberry fruis identify these three members (*FaERF3*, *FaERF6*, *FaERF71a*) as candidates to play a main role in the ripening receptacle. Their *in vivo* function, and the possible roles played in the crosstalk among hormones in this process, needs a further in depth study.

The separate analysis of achene and receptacle samples is also relevant for the assembly of the transcriptome. A high coverage is a critical factor for *de novo* assembly^18^. With the joint analysis of the ten samples there is the risk of losing transcripts abundant only in one of the samples, whereas the separate analysis would miss transcripts with low levels in all of the samples. Thus, ten separate assemblies were performed. Assembly of the transcriptome with Trinity and further clustering of the transcripts with the MIRA program resulted in 26,087 contigs, a number close to the 25,050 genes annotated in *F. vesca* ^7^, but lower than the 45,377 genes predicted in a virtual reference genome constructed from four homoeologous subgenomes of *F*. × *ananassa* ^2^. This probably means that in the MIRA contigs are included sequences from different alleles/homoeologs. The high percentage, over 85%, of them with high level of identity (Blastn < 1e-100) to two of the homoeologous subgenomes, *F. vesca* and *F. iinumae*, supports this possibility.

The information on polymorphisms in gene sequences is highly valuable for applications in breeding programs of cultivated strawberry because differential expression in particular alleles have been linked to phenotypes of interest^42^. Mapping the RNA-seq reads back onto these contigs using the FREEBAYES program allows the identification of polymorphisms in the transcriptome of the cultivated *F*. × *ananassa* species, indicating their relative abundance per tissue/sample. Its application to the *FaERF71a* gene, as an example, reveals the occurrence of a high level of polymorphism (21 positions), unequally distributed among the different tissues/samples. The result is somewhat expected because *F*. × *ananassa* is a highly heterozygous octoploid species. For a single *locus*, all of these polymorphic sites must be assembled in eight combinations, at most, because this is the highest number of possible alleles/homoeologs expected in an octoploid. This challenge cannot be solved yet using short Illumina reads, without a reference octoploid genome. In humans, emerging technologies, such as long-read sequencing (PacBio RNA-seq)^43^ and a microfluidics-based linked-read sequencing technology^44^, have successfully reconstructed full length transcripts and provided haplotype information at specific *loci*. However, particularly interesting in the case study of *FaERF71a* is the amino acid change at the AP2/ERF domain, which is composed of c. 60 amino acid residues with a well-defined conformation, that is responsible for the DNA binding in this family of TFs^27^. Any amino acid change might affect the biological activity of the protein encoded by this regulatory gene. The transcriptome datasets provided here make feasible this analysis for any expressed gene, and might contribute also to improve the annotation of strawberry gene models. We claim that the information released here, and the preliminary analysis performed in selected examples, will greatly assist researchers not only in studies of strawberry (*Fragaria* × *ananassa*), but also in RNA-seq studies of species with complex genomes.

## Methods

### Plant material

The strawberry (*Fragaria* × *ananassa* Duch. cv. Camarosa) fruits used for RNA-seq were harvested in four different developmental stages corresponding to green (G), white (W), turning (T) and red (R) (Fig. 1). The fruits were collected from plants that were grown under commercial field conditions in Huelva, Spain (37°14′27″N, 06°48′04 W), during early May. All of the fruits selected were phenotypically similar for each stage (*i.e*., shape, size and colour) and harvested at the same time (morning) of the day. All of the fruits were frozen in liquid nitrogen immediately after harvesting. Achenes and receptacle were separated from frozen fruits, to avoid changes in gene expression due to wounding, using a scalpel. After this process, a possible contamination of achenes from the receptacle (or vice versa) was assessed and cleaned under a magnifying glass. RNA for RNA-seq was extracted from three separate pools of samples totalling approximately 30 fruits each. Fruits from each pool were obtained from 10–15 plants. Young fully expanded leaves and actively growing roots were sampled in triplicate and frozen from the same plants grown under commercial conditions.

For the quantitative real time polymerase chain reaction (q-PCR) analysis, fruits from *F*. × *ananassa* cv. Chandler from plants growing in a greenhouse under natural light conditions (IFAPA-Churriana, Málaga, Spain, 36°40’51”N, 4°30’28”W) were harvested at three different developmental stages: green, white and red. Fruits were processed as previously described^38^. Analyses of the fruits were performed on three separate pools of 30 fruits for each ripening stage.

### RNA extraction and transcriptome analysis

Total RNA was extracted from strawberry achenes, receptacles, leaves and roots as previously reported^38^. RNA was treated with RNase-free DNase I (TURBO^TM^ Ambion, Invitrogen) according to the manufacturer’s instructions to remove contaminating genomic DNA. The RNA was quantified, and the quality was determined based on absorbance ratios at 260/280 nm and 260/230 nm using a NanoDrop spectrophotometer (NanoDrop Technologies). RNA integrity was assessed by electrophoresis under denaturing conditions and verified using the 2100 Bioanalyzer (Agilent, Folson), with RIN values above 8 for all of the biological replicates.

Expression analysis by RNA-seq of the developing fruit, leaves and roots was performed from three biological replicates. For each biological replicate, one paired-end library with an approximate 300 bp insert size was prepared using an in-house optimized Illumina protocol at Centro Nacional de Análisis Genómicos (CNAG, Barcelona, Spain). Libraries were sequenced on an Illumina HiSeq 2000 using 2 × 100 bp reads. More than 30 million reads were generated for each sample.

Gene expression by q-PCR was analysed using the fluorescent intercalating dye SsoFast EvaGreen supermix in the MyiQ detection system (Bio-Rad). In this analysis, first-strand cDNA synthesis was performed using 500 ng of RNA in a final volume of 20 μl using the iScript cDNA synthesis kit (Bio-Rad), according to the supplier’s protocol. Relative quantification of the target genes was performed using the comparative Ct method^38^. Expression data were normalized to the reference genes actin and glyceraldehyde-3-phosphate dehydrogenase 2 (*FaGAPDH2*). Primers used and PCR conditions are listed in Supplementary Table S3.

### Mapping the reads to the *F. vesca* reference genome and expression analysis

Raw reads were filtered to obtain high-quality processed reads by removing adapters, reads shorter than 50 bp, and low quality reads with Q-value ≤ 30, using Fastq-mcf from ea-utils^45^. Mapping to the reference genome, counting of reads, and analysis of gene differential expression and clustering were performed with “Tuxedo suit” (Hisat2/Cufflinks/CummRbund), using the default parameters^24^. The assembled *Fragaria vesca* genome (Fvb_genome) was used as a reference genome^3^. As an annotation reference, we used the RNA sequences from NCBI *Fragaria vesca* RefSeq Annotation Release 101 (Mar 2 2015) mapped on the Fvb genome with gmap^46^. Transcripts with normalized reads below 0.3 fragments per kilobase of exon per million fragments (FPKM) were considered as not expressed.

### *De novo* transcriptome assembly

The strawberry *de novo* transcriptome assembly was performed using Trinity software^21^ with the default parameters. Ten independent *de novo* assemblies, one for each sample, were obtained by gathering reads from the three replicates per sample. Isoforms resulting from the Trinity assembly were considered as putative transcripts for specific genes.

To reduce sequence complexity of the assemblies, isoforms resulting from the Trinity program were first filtered by expression values (>0.3 FPKM) and then by BLASTX hits (1e-30) with the UniProt Plants Database. Further assembly of sequences into contigs was performed with the MIRA program^30^. The quality of these contigs was assessed by mapping the RNA-seq reads back onto the assembled contigs. Then, a homology search of contigs was performed by BlastN (p < 1e-100) against the NCBI *Fragaria vesca* RefSeq annotation and *Fragaria iinumae* predicted genes^2^. The variants in the bam files generated in the mapping-back process were called with the FREEBAYES program^47^.

### Data mining, graphical representation and statistical analysis

Matrix data mining, normalization, clustering (hclust, “stats” package^48^), and graphical representation were performed using R software^48,49^. The CummRbund v2.16.0 package^50^ was also used.

For the graphical visualization on diagrams of differentially expressed genes (DGE) between different tissues and stages, in the context of different metabolic pathways and other processes, we used Mapman 3.6.0RC1^51^ stablishing a correspondence between the putative *loci* obtained from Cufflinks and their significant BLASTN homologues in the *F. vesca* gene model 1.1^7^ and the associated mapping file F. vesca226 from Phytozome 9.0.

All bioinformatics processes were developed at The Supercomputing and Bioinnovation Center of the University of Malaga (http://scbi.uma.es). Reads and processed files are stored at the European Nucleotide Archive (https://www.ebi.ac.uk/ena) with the study reference PRJEB12420.

## Electronic supplementary material

Supplementary Information

File_S1

File_S2-7_and_ Suppl_Tables_1-3. File  S8 and File S9.
