# Supplementary material for: Gene expression atlas of fruit ripening and transcriptome assembly from RNA-seq data in octoploid strawberry (Fragaria × ananassa)
Source: Sci Rep. 2017 Oct 23;7:13737. doi: 10.1038/s41598-017-14239-6 (PMC5653846; doi:10.1038/s41598-017-14239-6)
Supplement: Supplementary file 1 — Supplementary Information [file 41598_2017_14239_MOESM1_ESM.pdf]

Supplementary Information

Gene expression atlas of fruit ripening and transcriptome assembly from RNA-seq data in octoploid strawberry (Fragaria × ananassa)

José F. Sánchez-Sevilla, José G. Vallarino, Sonia Osorio, Aureliano Bombarely, David Posé, Catharina Merchante, Miguel A. Botella, Iraida Amaya, Victoriano Valpuesta

Supplementary File S1. Expression values, as FPKM, corresponding to the 28,574 XLOCs as described in Figure 2.

Supplementary File S2. Table of the Fragaria vesca genes, identified by MapMan analysis, corresponding to the Fragaria x ananassa ethylene-related genes differentially expressed in achene in the transition from green (G) to white (W), as shown in Figure 4.

Supplementary File S3. Table of the Fragaria vesca genes, identified by MapMan analysis, corresponding to the Fragaria x ananassa ethylene-related genes differentially expressed in achene in the transition from white (W) to turning (T), as shown in Figure 4.

Supplementary File S4. Table of the Fragaria vesca genes, identified by MapMan analysis, corresponding to the Fragaria x ananassa ethylene-related genes differentially expressed in achene in the transition from turning (T) to red (R), as shown in Figure 4.

Supplementary File S5. Table of the Fragaria vesca genes, identified by MapMan analysis, corresponding to the Fragaria x ananassa ethylene-related genes differentially expressed in receptacle in the transition from green (G) to white (W), as shown in Figure 4.

Supplementary File S6. Table of the Fragaria vesca genes, identified by MapMan analysis, corresponding to the Fragaria x ananassa ethylene-related genes differentially expressed in receptacle in the transition from white (W) to turning (T), as shown in Figure 4.

Supplementary File S7. Table of the Fragaria vesca genes, identified by MapMan analysis, corresponding to the Fragaria x ananassa ethylene-related genes differentially expressed in receptacle in the transition from turning (T) to red (R), as shown in Figure 4.

Supplementary Files S8. Trinity isoforms for the 10 RNAseq samples are available d at www.ebi.ac.uk/ena, ref. PRJEB12420.RNASEQ, as:
Transcriptome shotgun assembly (TSA) – UNANOTATED :HX2000058865

Transcriptome shotgun assembly (TSA) – UNANOTATED :HX2000058865
Transcriptome shotgun assembly (TSA) – UNANOTATED :HX2000058864
Transcriptome shotgun assembly (TSA) – UNANOTATED :HX2000058863
Transcriptome shotgun assembly (TSA) – UNANOTATED :HX2000058862
Transcriptome shotgun assembly (TSA) – UNANOTATED :HX2000058861
Transcriptome shotgun assembly (TSA) – UNANOTATED :HX2000058860
Transcriptome shotgun assembly (TSA) – UNANOTATED :HX2000058859
Transcriptome shotgun assembly (TSA) – UNANOTATED :HX2000058858
Transcriptome shotgun assembly (TSA) – UNANOTATED :HX2000058856
Transcriptome shotgun assembly (TSA) – UNANOTATED :HX2000058854

Supplementary File S9. MIRA contigs, are available at www.ebi.ac.uk/ena, ref. PRJEB12420.RNASEQ, as Transcriptome shotgun assembly (TSA) – UNANOTATED : HX2000059175

Supplementary figure S1: HeatMap of the Metabolism Overview corresponding to the DEGs (File S1) in the successive transitions from green (G), white (W), turning (T) and red (R), in the achene (A) and the receptacle (R), obtained from the MapMan analysis. Abbreviations of the pathways/cycles are CHO: carbohydrate: OPP: oxidative pentose phosphate; TCA: tricarboxylic acid. The color key indicates the log2 fold change between the transitions.

Green Achene - White Achene

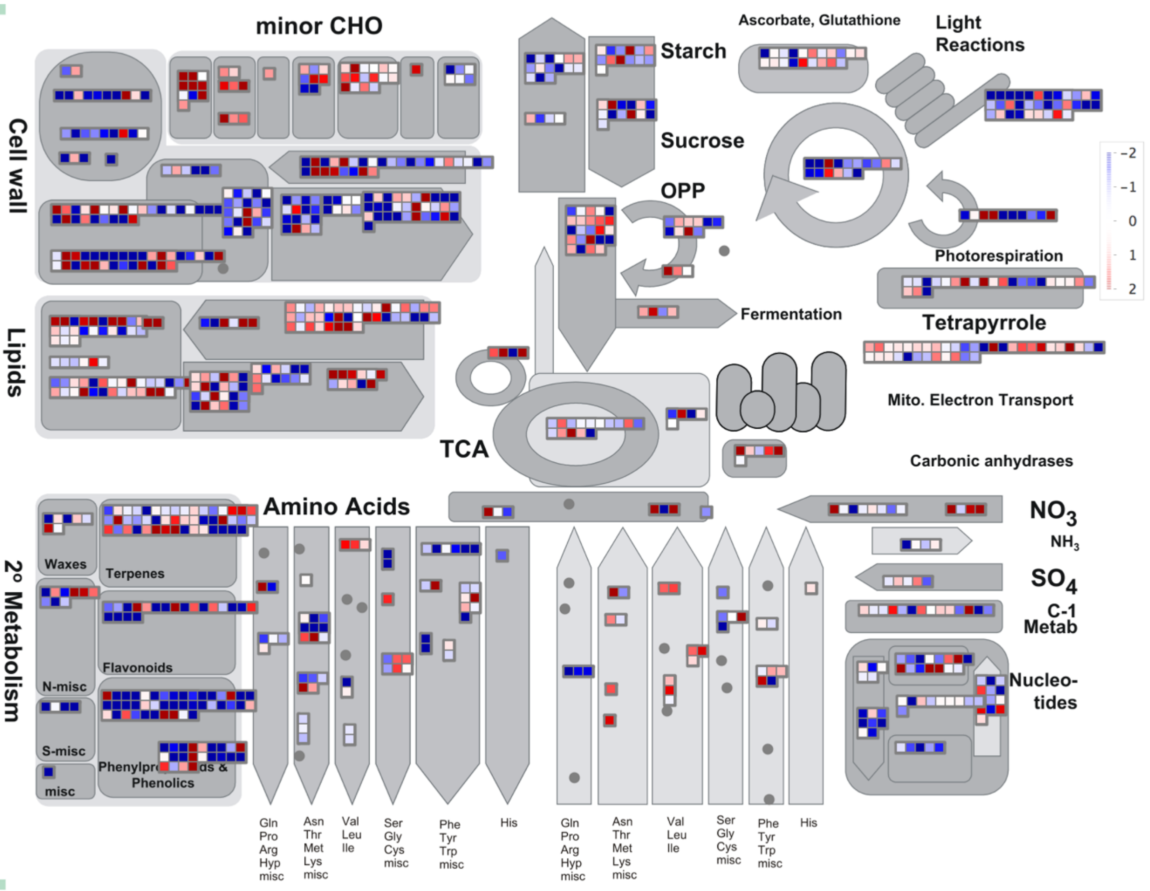

White Achene - Turning Achene

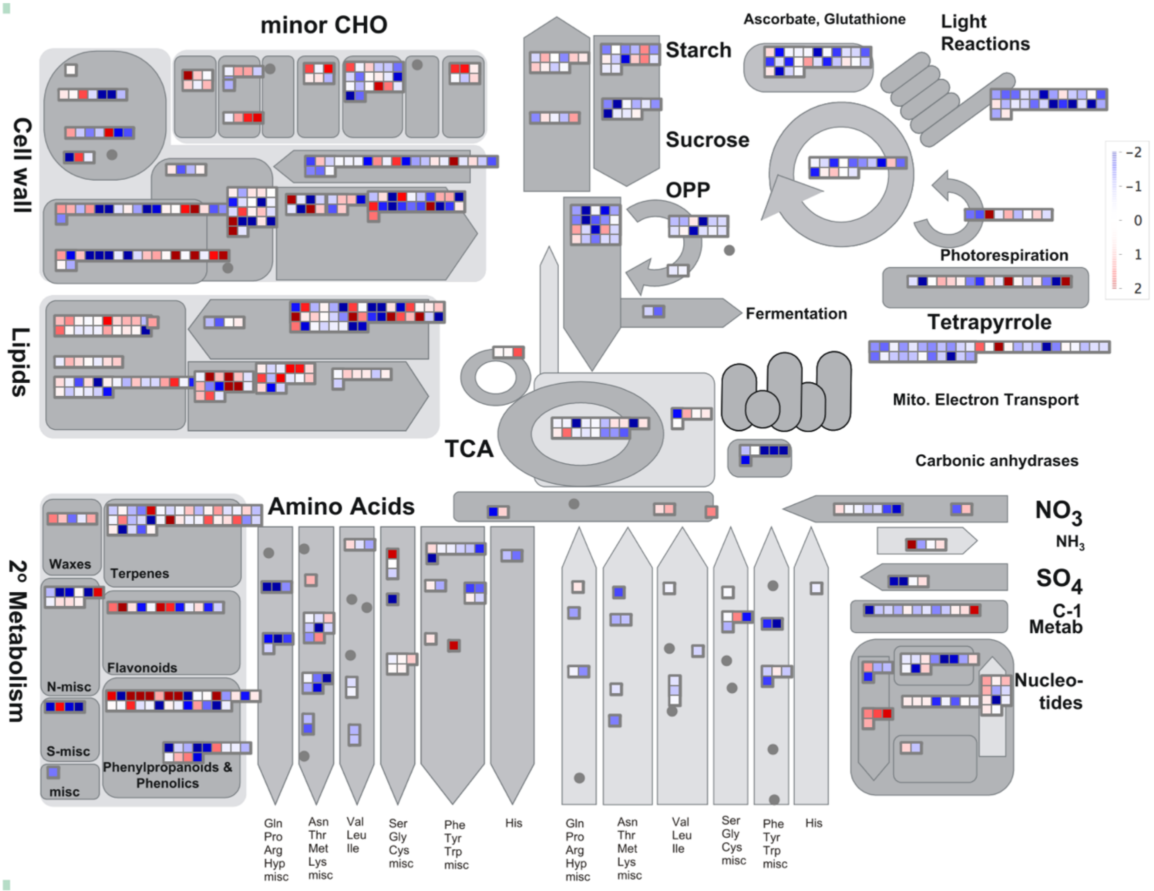

Turning Achene - Red Achene

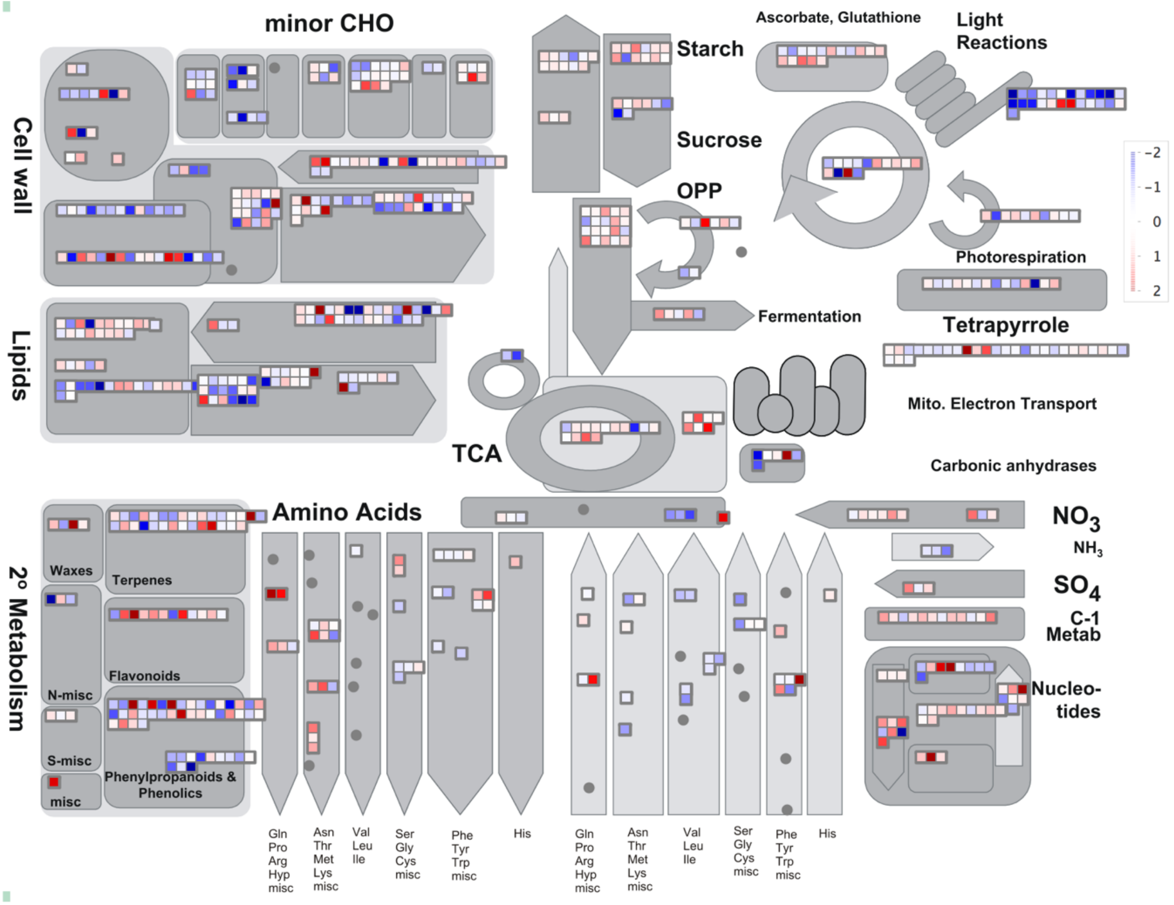

Green Receptacle - White Receptacle

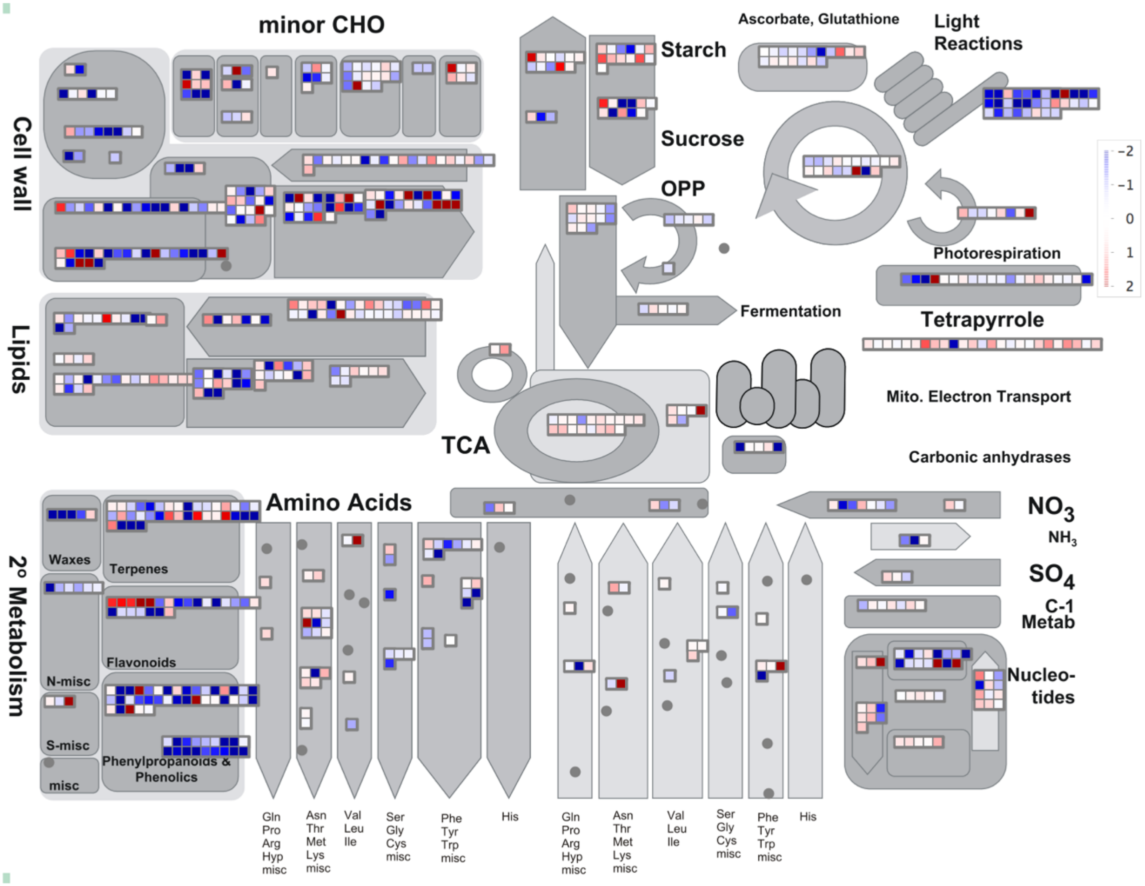

White Receptacle - Turning Receptacle

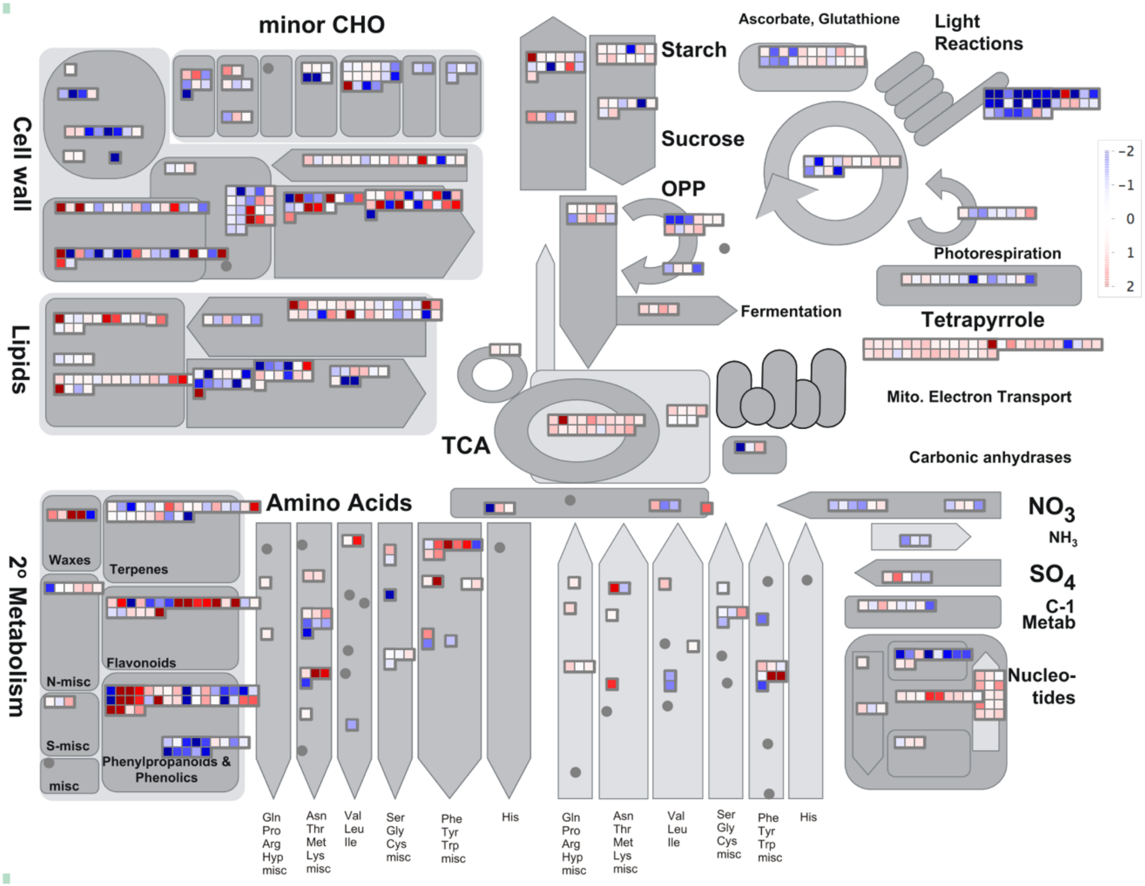

Turning Receptacle - Red Receptacle

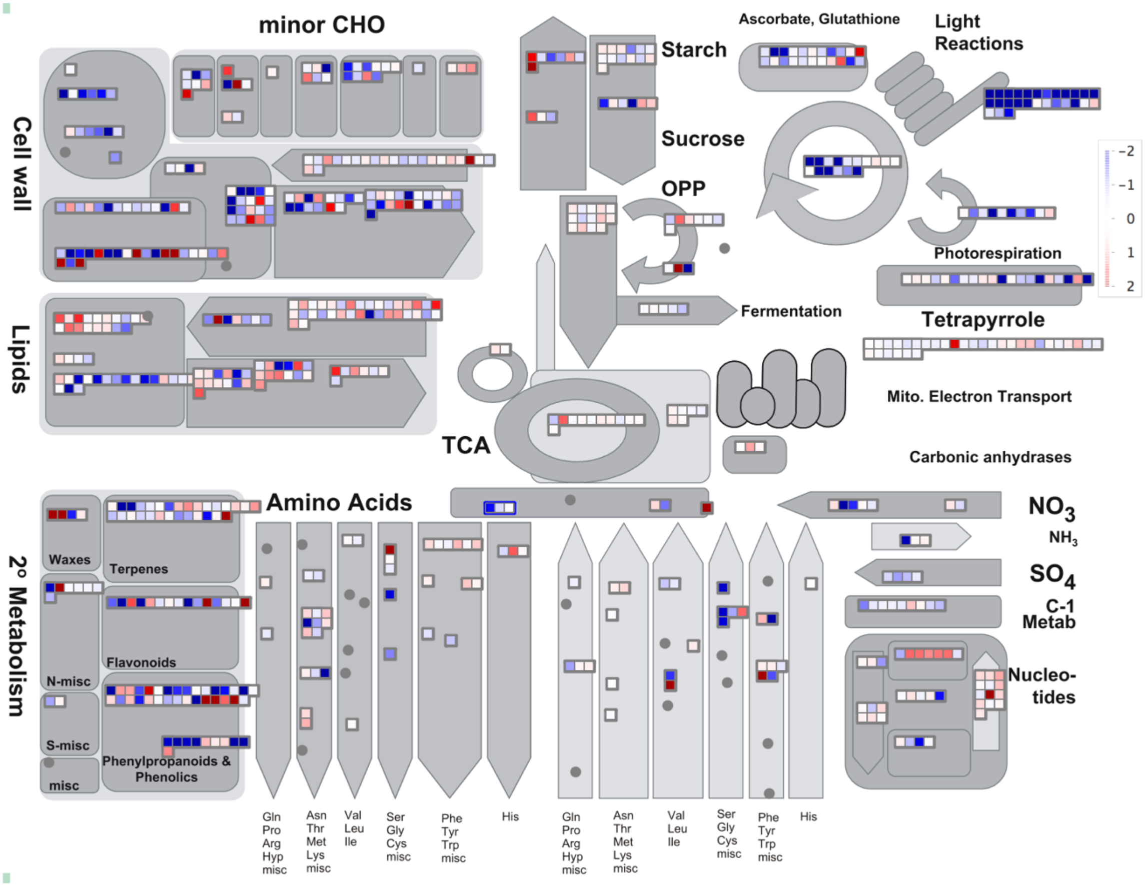

Supplementary figure S2: Dendrogram of the alignment of the mapped *Fragaria vesca* ERF genes with putative orthologues in *Arabidopsis.*

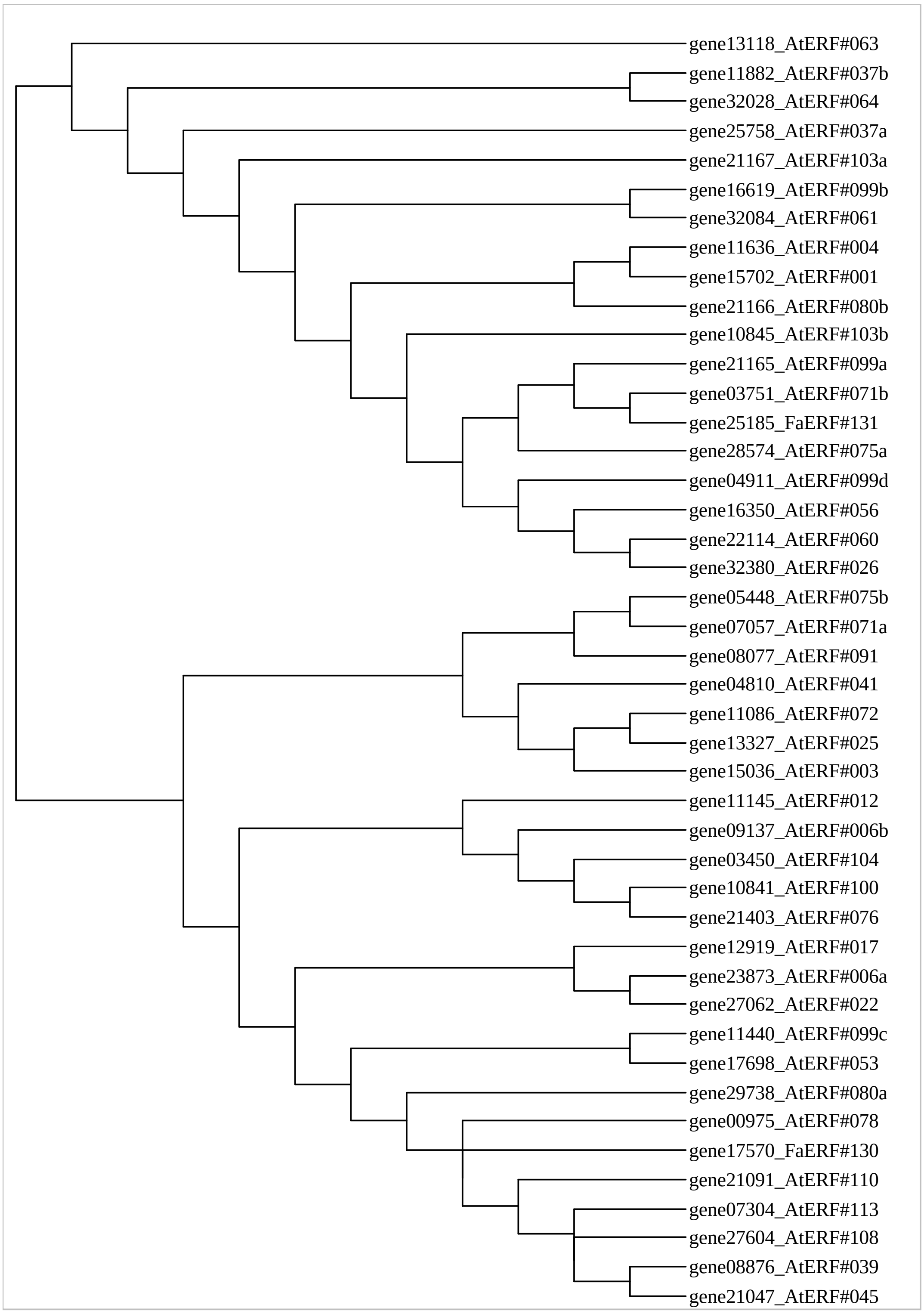

Supplementary table S1: Summary of the mapping of the RNA seq reads on the *Fragaria vesca* genome (Fvb_genome).

Supplementary table S2: Summary of the mapping back of the RNA seq reads on the contigs assembled with the MIRA program, from the Trinity isoforms.

Supplementary table S3: List of the primer and conditions used for quantitative PCR assays.
